# Electrically Doped Nanoscale Devices Using First-Principle Approach: A Comprehensive Survey

**DOI:** 10.1186/s11671-020-03467-x

**Published:** 2021-01-29

**Authors:** Debarati Dey, Debashis De, Ali Ahmadian, Ferial Ghaemi, Norazak Senu

**Affiliations:** 1 Department of Electronics and Communication Engineering, B. P. Poddar Institute of Management and Technology, 137, V. I. P Road, Kolkata, West Bengal 700 052 India; 2 Department of Computer Science and Engineering, Maulana Abul Kalam Azad University of Technology, NH-12(Old NH-34), Haringhata, Post Office – Simhat, P.S. – Haringhata, Pin – 741249, Kolkata, West Bengal 700 064 India; 3 Department of Physics, University of Western Australia, M013, 35 Stirling Highway, Crawley, Perth, WA 6009 Australia; 4 Institute of IR 4.0, The National University of Malaysia (UKM), 43600 Bangi, Selangor Malaysia; 5 Department of Chemical and Process Engineering, Faculty of Engineering and Built Environment, Universiti Kebangsaan Malaysia (UKM), Bangi, Selangor Malaysia; 6 Institute for Mathematical Research (INSPEM), Universiti Putra Malaysia (UPM), 43400 Serdang, Malaysia

**Keywords:** Electrical doping, DFT, NEGF, First principle, Molecular modeling

## Abstract

Doping is the key feature in semiconductor device fabrication. Many strategies have been discovered for controlling doping in the area of semiconductor physics during the past few decades. Electrical doping is a promising strategy that is used for effective tuning of the charge populations, electronic properties, and transmission properties. This doping process reduces the risk of high temperature, contamination of foreign particles. Significant experimental and theoretical efforts are demonstrated to study the characteristics of electrical doping during the past few decades. In this article, we first briefly review the historical roadmap of electrical doping. Secondly, we will discuss electrical doping at the molecular level. Thus, we will review some experimental works at the molecular level along with we review a variety of research works that are performed based on electrical doping. Then we figure out importance of electrical doping and its importance. Furthermore, we describe the methods of electrical doping. Finally, we conclude with a brief comparative study between electrical and conventional doping methods.

## Introduction

Doping plays a crucial role in determining physical characteristics and their applications of various organic or inorganic materials, especially for semiconductors. This method has been successfully proved for the semiconductor physics industry. A small amount of addition of impurities determines the dopant concentration and electrical conductivities of the materials. It is observed that an ideal dopant should exhibit an ideal solubility in its host material, and it also exhibits a low defect level. However, some basic problems are related to this type of conventional doping process, for example, doping bottleneck which powerfully affects the device performance. This type of performance degradation has been observed severely for wide bandgap materials.

For example, in the case of the minima of high conduction band device, n-type doping is challenging, whereas for maxima of the low valence band device is also complicated [1, 2]. Therefore, some problems arise for the bipolar doping process in wideband semiconductors. It is observed that either p-type or n-type dopants can be inserted but not together [3]. Therefore, to compensate for this type of problem, a feasible solution has been incorporated into the domain of doping. This type of proposed approach is known as electrical doping, which does not depend on this type of bipolar doping. Electrical doping has been introduced to solve the problems of bipolar doping. In the late 1980s’ and 1990s’, researchers observed that III–V compounds like a single crystal of GaN are difficult to grow. Even more, for commercial use of GaN substrates were also unavailable at the era of the late 1990s’. The reason behind it was explained in such a manner that the difference between lattice constants and coefficients of thermal expansion of the sapphire substrate and the GaN semiconductor made it difficult to grow a high-quality GaN-based epitaxial layer on the sapphire substrate. On the other hand, it was almost impossible to obtain a p-type GaN semiconductor due to combinations of high n-type background concentration and low p-type doping activity. This problem can be significantly overcome using the electrical doping phenomenon by Rudaz in the year of 1998. During the late 1980s’, scientists discovered the importance of growing GaN or AlN buffer layers to demonstrate GaN-based LED at low temperatures. Post-growth thermal annealing process helps to activate the growth of p-type dopants in GaN buffer layers. These advancements accelerated the growth in device development of the III–V nitride semiconductor material system for wide-band optoelectronic devices [4]. GaN substrate and post-growth thermal annealing process also play an important role in this technique [5–7]. Since last few decades, plasma etching technology plays an important role in ultra-large-scale technology (ULSI) to shrink the pattern size. This led us to the evolution of nanotechnology. On the same time, plasma technology faced some inherent problems for example; build up of charge, photon UV radiation along with etching performance for nanoscale devices. To remove these problems and fabricate practical nanoscale devices, neutral–beam etching process has come into the field. S. Samukawa has introduced this neutral beam sources and also talked about the combination of top-down and bottom-up processing for prospect nanoscale devices. Neutral beams technology is executed damage-free etching because it is used atomically. Using this technique surface modification of inorganic and organic materials can also be done. This technique is a capable contender for the practical fabrication technology for future nanodevices [8]. This high-density plasma technology includes inductively coupled plasma (ICP) and electron-cyclotron-resonance (ECR) plasma, which are the key methods for the implementations of this plasma technique. But there are several problems associated with this technique, such as

- Various types of radiation may damage the charge buildup of positive ions and electrons [8–12].
- The radiation of ultraviolet (UV), vacuum ultraviolet (VUV) ray may also damage nanoscale devices.
- X-ray photons may also cause rupture of nanoscale devices during this plasma etching problem [13–21].
- Due to charge buildup due to voltage generation distort ion trajectories, it also leads to fracture of thin gate oxide films.
- In addition to these, UV or VUV photons radiating from the high-density plasma etching technique lead to generate crystal defects.

These problems strongly degrade electrical properties of nanoscale devices. Therefore, these problems can be avoided using high-performance neutral-beam etching system. S. Samukawa and his group have invented a highly efficient neutral-beam source to realize the ultimate top-down etching for future nanoscale devices. They introduced the ultimate etching processes for future nanoscale devices from 50 nm to sub-10 nm in using our new neutral beam sources.

This letter is therefore organized as follows. Firstly, the historical roadmap of electrical doping is briefly reviewed. After that, we will review some experimental work at the molecular level as this doping process has its impact on the molecular level, too. Then, we give brief discussions on a variety of research works associated with the electrical doping process. Some of the importances of electrical doping are described in the following section. Furthermore, we describe the method of the electrical doping process. Finally, we will conclude with the brief discussion of the comparative study between conventional doping and electrical doping.

## Historical Roadmap of Electrical Doping

Although this study mostly concentrates on electrical doping at the molecular level, it is important to first review the early history of conventional doping. In the year of 1930, it was noticed that the conductivity of semiconductors was affected due to the presence of a little number of impurities [2, 22, 23]. In the year 1931, the first quantum–mechanical formalism was used for semiconducting materials [24]. The prototype of a p–n junction was successfully demonstrated by Davydov in the year of 1938 [25, 26]. This article explained the importance of minority carriers. Woodyard introduced the concept of “doping.” He incorporated a small portion of phosphorus, arsenic or antimony into pure germanium. This addition of impurity increases the electrical properties of germanium [27]. Shockley proposed his historical invention, i.e., “junction transistor” in the year of 1949. This invention changes the geometry of the semiconductor industry [28]. Though the invention of bipolar junction made a tsunami to the evolution in the semiconductor industry, it had several problems too related to transistors. For example, two p–n layers should be junction together back to back within a thin space. This problem was removed after the invention of “grown junction transistor” at Bell laboratory in the year 1950 using a double-doping method [29, 30]. In the case of the “double-doping” process, a pinch of gallium was added into the molten n-type germanium, which transformed germanium into p-type. Afterward, a pinch of antimony was included to it which transforms it to p-type back to n-type [31]. Two types of dopants were added back to back in this process. There is another kind of doping that was evolved in the early 1950s, which is known as “co-doping.” The p and n junctions are considered as “co-doping” of a semiconductor. The doping at the molecular level is also an important part of electrical doping. In the year of 1998, Rudaz proposed a method to maximize the effect of electrical doping by reducing material cracking for III–V semiconductors [4]. In the year of 2002, Zhou et al. demonstrated vacuum-deposited transparent organic light-emitting diode which is also a low-voltage device by using the electrical doping process. Electrical doping plays a crucial role to improve the performance of organic devices. Electrically doped carrier injection takes place for organic LEDs (OLEDs). The transport layers show low driving voltages, which is generally due to the radical anions, cations and ohmic contacts at the end of the electrode interfaces. Ultra-low-voltage OLEDs are vacuum deposited with 2.6 V for 100 cd/m^2^ in p–i–n structure. Therefore, an intrinsic emission is sandwiched between p- and n-type wide bandgap transport layer. The activities related to electrical doping in organic molecular films are emphasized in a few studies [32–42].

This is one of the procedures to avoid the ionic bombardment process in the atomic-scale device designing approach. Gao and Kahn [43] have demonstrated this process onto the molecular thin films. These compounds for example poly-carbonate polymer with tris(4-bromophenyl)aminium hexachloroantimonate (TBAHA) 4,4′,4″-tris(3-methylphenylphenylamino)-triphenylamine (m-MTDATA) hole transport layer p doped with F4-TCNQ are used to successfully fabricate various OLEDs compound device layer [45, 46]. This process has also been used in organic photovoltaic cell (OPVC). This process has also been used for tuning at the molecular level and also for improving the device efficiency enhancement by carrier injection. Molecular film conductivity increases to a large extent for n- and p-type doping by using this process. This doping process is extensively used for ohmic contacts on inorganic semiconductors [43–46]. Nowadays, organic LEDs hold effective footage in the field of molecular nanotechnology. In III–V semiconductor, using this doping process n-type contacts and insertion of n-type external molecules can be made possible. Electrical doping also helps to make possible the phenomenon like electrical resistance, carrier insertion, carrier recombination into the molecular interfacing layer. Organic photovoltaic cell (OPVC) is one of the most relevant applications of the electrical doping phenomenon. In the process of level alignment for OPVC, this process acts on the conductivity of these cells. Charge carrier insertion is eventually increased by this method. In the case of meta-organic interfaces, this method begins and brings to the arrangement of a depletion layer through which quantum tunneling transmission can take place. This is one of the efficient processes which can be effectively used for organic and inorganic contact fabrication. This process also helps to shift the charge neutrality levels for molecular thin films. In addition to this, approximately 0.1 to 1% of foreign molecules can be included using this method to the molecular interfaces. This amount of doping concentration is a large number for the conventional doping method. This level of doping concentration helps to generate de-generates semiconductors. This high doping concentration helps to prevent the subsequent formation of doing-induced bands [34, 43–46].

## Electrical Doping Process and Its Importance

The main and foremost technique opted for the electrical doping method is to control the Fermi level using this process. Therefore, this technique is highly popular among inorganic and organic semiconductors for the past few decades. Electrical doping in recent years has attracted special attention in the field of bioinspired nanotechnology. Electrical doping is the process of electronic charge insertion or acceptance of them to molecular films. The key feature of this process is that the conventional n and p doping cannot be constrained to accomplish the bipolarity. The conventional ionization process is not applied for this type of electrical doping process [43–46]. The electrical doping procedure has been introduced to avoid ionic bombardment, which is generally not possible for nanoscale device modeling.

This method of doping was determined mainly in two steps:

- The first step is involved with a single electron transfer from donor to an acceptor (into molecules).
- Secondly, it is associated with the method of dissociation of ground state integer charge-transfer complex.

Thus, it is confirmed that electrical doping is nothing but to shift of the Fermi level either toward the highest occupied (valence band) molecular level or lowest unoccupied (conduction band) molecular state. If the free carrier is *ρ*, *N*_A_^−^ is ionized dopant density, *N*_A_ is the neutral dopant concentration, then the free carrier density is to be formulated as in Eq. (1). In this equation, *E*_A_ and *E*_F_ are the acceptor and Fermi-level energies and *K*_B_ is the Boltzmann constant at absolute temperature *T* [124].

1 $$\rho = N_{{\text{A}}}^{ - } = \frac{{N_{{\text{A}}} }}{{1 + \exp \left( {\frac{{E_{{\text{A}}} - E_{{\text{F}}} }}{{K_{{\text{B}}} T}}} \right)}}$$

This doping technique has been carried out using two-probe design techniques in Atomistix Tool Kit-Virtual Nano Lab (ATK-VNL). The number of bioinspired atomistic devices lies at the heart of nanotechnology. These devices operate at ultra-high THz frequency. The frequency that is calculated for these device is around THz. For example in a article, where transport characteristics for GaAs-Adenine-GaAs semiconductor tunnel junction is illustrated. In that article the operating frequency is reported about 25THz [125].

Doping is an intentional induction of external impurities into a pure semiconductor material for the reason of enhanced electrical performances. Importance of electrical doping process can be described as follows.

This electrical doping procedure is different from the conventional doping process. In the case of the conventional doping process, the semiconductor material is doped with extrinsic dopants or impurity. This process is the high-temperature process. There is a chance of breakage of bonds that may happen during this high-temperature doping process. The ionization method is also adopted to implement this doping method. On the other hand, the electrical doping process is not related to impurities at all. As in this procedure, opposite potential charges are induced at the two ends of the device. Therefore, it will generate a potential drop in the central molecular region of the nanodevice. This method is quite helpful for nanodevice designing because the ionization method may generate structural deformation for the nanomaterials. In the case of conventional doping, several problems may arise. Some of the major problems are listed in Table 1. Differentiation between conventional and electrical doping is framed in Table 1, and it also helps to understand how electrical doping is important for nanoscale device fabrication.

**Table 1:** Difference between conventional doping and electrical doping

Conventional doping	Electrical doping
A little amount of interaction is observed between dopant and host atom	There is no chance of interaction between host and dopant atoms
Mismatching of compatibility between foreign atoms and host atoms	No probability of compatibility mismatching as it depends on bias voltage
The exothermic reaction may take place	There is no chance of heat generation. Because heat may destroy molecular thin films
The intermolecular reaction may be observed during this process	No intermolecular reaction takes place

This Table 1 shows why electrical doping is important for molecular level. This doping avoids the heat generation, interatomic or intermolecular reaction along with it is compatible for any kind of nanoscale device designing procedure.

**Fig. 1:**
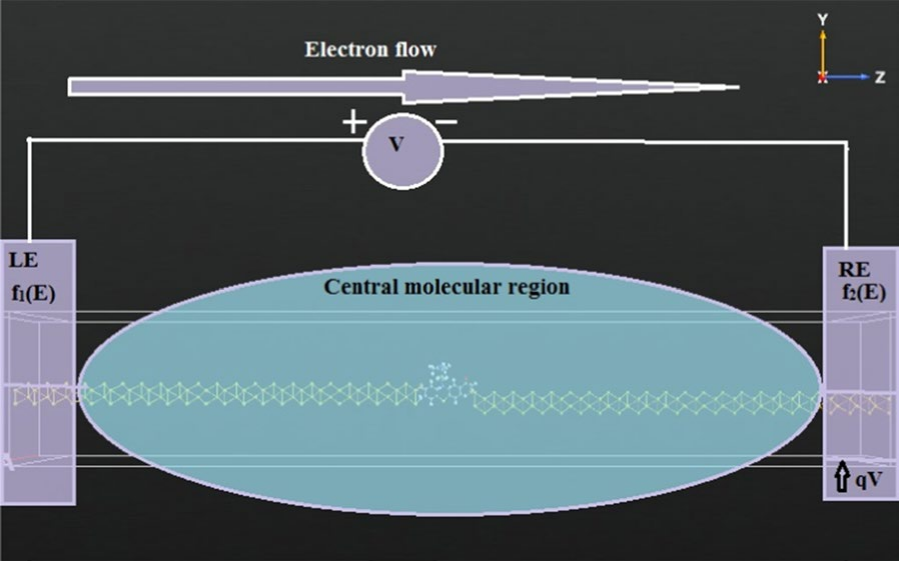
Schematic diagram of the conceptual electrical doping process

In this article, electrical doping process is mainly highlighted. This doping method is useful for nanoscale device fabrication mainly molecular thin film preparation. In this method insertion of charge carriers takes place at the two ends of the molecular device. This process is also depicted in Fig. 1. This diagram represents the simple electrical doping method. This figure also shows how potential drop has been created due to the insertion of two equal but opposite charge carrier insertion at the two terminals of electrodes. These electrodes are the important part of molecular device. The charge insertion can be done through these electrodes. This equal and opposite charge creates a potential drop within the central molecular region. This potential drop acts as the driving force of charge conduction between two electrodes, i.e., through the central molecular part. This is the actual process of electrical doping. Though this process is nowadays used in analytical or theoretical modeling of nanoscale devices mainly, also it is useful for organic and inorganic molecular thin film preparation.

Figure 1 shows how electron or charge carrier flows from one electrode to another direction due to the potential drop that has been imposed due to variation of bias at the two terminals of electrodes.

## Electrical Doping at the Molecular Level

Recently researchers are interested for controlled doping procedure. Therefore, this electrical doping procedure helps to introduce controlled doping for inorganic semiconductors. Thus, it is also helpful to tune electrical properties of these semiconductors by introducing electrical doping. This doping phenomenon helps to tune optical gap of semiconductors with their chemical variation. This doping procedure is also a low-cost process and useful for flexible substrates.

**Fig. 2:**
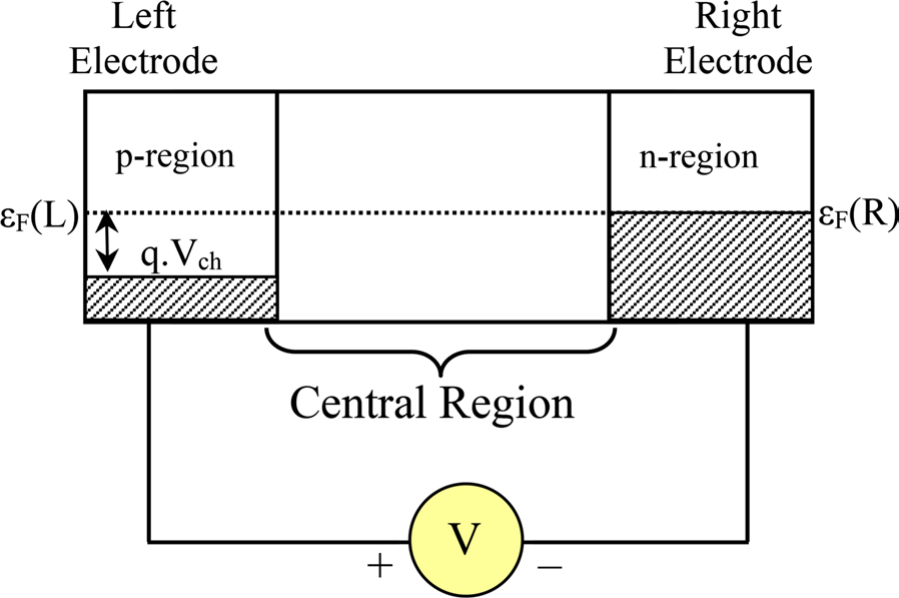
Schematic diagram of the conceptual electrical doping process (using ATK-VNL)

The electrical doping procedure is the method by which a potential difference has been created between the two ends of the nanodevice. In this theoretical work [47–52] we have arranged this by providing different polarity but same-valued voltage at the two ends of the nanodevice via two-probe electrodes. The schematic diagram for this theoretical process is shown in Fig. 2.

This theoretical approach is involved to create highly doped positive (p+) and negative (n+) regions, which are important to design nanosemiconductor devices for both organic and inorganic materials.

Using this procedure charge carriers are to be injected into the molecular interfaces. Electrical doping is a controlled process for organic molecules rather than inorganic thin films. Therefore, conventional p and n dopants are not mandatory for insertion. Eventually, electrical doping increases carrier injection and decreases drive voltage which leads to a rise in device efficacy. Thus, electrical doping method solely depends on the injection of either electronic transmission or electron reception to the host molecule.

The heterojunction chain is made with adenine and thymine biomolecules used to sense several gases when the chain passed through the nanopore of a GaAs nanosheet [47]. In that case, also the electrical doping is induced at the two parts of this nanosheet. Due to the effective inductance, this biomolecular chain shows its ability to sense the adsorbed foreign gas molecules [47]. In the case of nanodevice design is also dealt with adsorption of molecules. For example, adsorption of volatile molecules at 32 °C temperature into ZnO nanowire is investigated [53]. Using DFT and NEGF formalisms-based first-principle approach, nano-FET can be designed using various structural modifications. Various properties of these nano-FETs’ are also observed, for example, scalability assessment, highest occupied molecular orbital–lowest unoccupied molecular orbital (HOMO–LUMO) gaps, maximum obtainable current, RF performance, linearity investigation [54–61]. Conjugated co-oligomers-based molecular diode can be designed using DFT- and NEGF-based formalisms. The co-oligomers are connected with two electrodes and form a molecular diode. The energy gap, current–voltage (I–V) characteristics, spatial orientations are analyzed for this diode [62]. The first-principle approach is applied to the geometrically optimized nanostructures of seven different junctions which are derived from carbon nanotube (CNT) using different linkers [63]. Various types of diodes can be implemented using DFT and NEGF formulas-based first-principle approach. For example, Schottky diode, single molecular diode, spin current diode, bipolar spin diode, di-block molecular diode, backward diode characteristics are therefore implemented using this approach [64–68].

## Molecular-Level Research Works Based on Electrical Doping

Electrical doping at molecular level plays an important role in nanoelectronics. Researchers are highly interested to introduce this doping procedure at nanoscale device designing procedure. The effect of this doping helps to interface between different molecular level of alignment. This process is not only helpful to study organic heterojunction molecular level but also acceptable for inorganic materials. This doping helps for the interface formation with the help of dipole and equivalent move in comparative position of molecular interface. Thus, this process of electrical doping is acceptable for molecular interface alignment.

Miniaturization of conventional electronic devices is the most emerging research area nowadays. There are several approaches which lead to motivate researchers to investigate and study the nature of nanoscale devices. One of the most important approaches is to design and simulate analytical nanostructures. Many significant devices can be designed using this simulation procedure and analyze the obtained results [47, 55, 56]. According to the result, the researchers can modify the various simulation parameters as well as the different aspects of the nanoscale analytical model. Among these simulation methods, the first-principle approach is the most effective and popular process. Modernization of electronic devices encourages researchers to innovate conventional devices in a modified version. For example, traditional semiconductor devices can be designed using biomolecules. In the case of biomolecules generally, nucleobases such as adenine, thymine, guanine and cytosine have been considered which are known as the basic building blocks of DNA [47, 55]. It is very common to construct conventional inorganic semiconductor devices in the field of nanotechnology. However, it is hard to construct organic electronic devices mainly using biomolecules. These semiconductors are characterized depending upon the doping properties. If the semiconductor does not have any impurity doping, then it is called intrinsic or pure semiconductor. On the other hand, if the semiconductor is doped with foreign atoms or molecules, then it is known as an extrinsic or impure semiconductor [55–60].

Nowadays, nanoscale device designing is a challenging aspect for researchers. Diode, transistor, logic gates have already been implemented at the molecular level. There is another scope for researchers to implement nanobiosemiconductor devices at the molecular level. Some of these biomolecular devices have already been introduced in the arena of biomedicine. The theoretical design of these nanodevices has been implemented using the Atomistix-Tool Kit and Virtual Nano Laboratory (ATK-VNL)-based Quantumwise software simulator version 13.8.0 [69–76]. Even Quantum Cellular Automata (QCA) logic can be theoretically implemented using DFT and NEGF-based first-principle approach [77]. Various logic gates can be made possible to design using biomolecules, and the results obtained from these theoretical implications have also validated using Multi-Sim or SPICE or other simulators [70]. The electrical doping process is the key feature that is introduced to obtain optimum current. Tunnel current through the molecular channel is affected by various factors like back-scattering effect, etc. By implementing this doping process, we can avoid the problems related to the conventional doping process. The dipole combination model for Schottky barrier tuning is also suggested at the metal–semiconductor interface at the molecular level [78]. The first-principle approach is also applicable for magnetic tunnel junction, and their quantum electronic properties have been analyzed [79]. To calculate leakage current through SiO_2_ and SiO_x_N_y_-based MOSFET, researchers used DFT- and NEGF-based first-principle approach [80]. This ab-initio modeling is applied for modeling of Schottky barrier height tuning using the yttrium and nickel silicide atomic-scale interface [81]. Direct band to band tunneling in reverse-biased MOS2 p–n junction nanoribbon can be described using DFT and NEGF [82]. The effect of incorporation of opposite polarities dopant atoms into the nanowire exhibits electrical properties like Zener diode [83]. The dual-spin filtering effect can be seen in the half-metallic yttrium nitrite YN_2_ [84]. Investigation of heterostructure biomolecular FET can be observed using this electrical doping technique. The quantum ballistic transport can be observed using this electrical doping phenomenon at the molecular level [85]. Using this theoretical approach electrically doped biomolecular switch is designed when using single-wall carbon nanotube (SWCNT) as electrodes [86]. NEGF formalisms help to design graphene-based anti-dot resonant tunnel diode [87]. Atomistic characteristics of two-dimensional silicon p–n junctions have been demonstrated using the first-principle approach [88]. Diode and transistors are the basic building blocks of any electronic circuitry. Logic gates can also be implemented using diodes and transistors. Therefore, any logic can be implemented using first-principle formalisms.

In the recent trend of nanotechnology, researchers have interested to design and characterize the various electromechanical features of bioinspired and semiconductor devices at the atomic scale. These bioinspired devices are highly biocompatible and create a bridge between the semiconducting area and the bi-molecular research arena. CMOS technology has been already saturated. Therefore, the aim of the researchers is to replace and create a bridge between them. Several proposals have already been raised by the researchers to join the CMOS technology with bioinspired technology like DNA or any other biomolecules. The important parts of the DNA are adenine, thymine, cytosine and guanine nitrogen bases. These nitrogen bases have made composites with ribose sugar and phosphate groups to form oligonucleotide. This oligonucleotide has phosphate groups as the backbone. Correlations for dynamic signals have been enhanced for the identification of biomolecules and DNA [89]. DNA translocation, electronic transmission and semiempirical modeling through graphene nanopore can also be made possible theoretically using DFT and NEGF [90–93]. DNA analysis can also be made possible with graphene electrodes using semiempirical modeling [94]. Recognizing of nucleic acid base pairs using transverse transport properties has also been made possible [95]. Conductance through shot DNA has also been proposed by the group of researchers [96]. Electronic enhancement by doping procedure to the DNA base pairs has also been incorporated to enhance the conductivity [97]. The electronic promotion has also been possible by the double-proton transfer process [98]. Recognition of nucleotides by the cross-tunneling method has also been possible using the first-principle approach [99]. Structural factors control the conductivity of DNA, and this has also been discussed in [100]. The nanoscale devices exhibit an enormous quantum transport phenomenon for different types of nanoscale device modeling [56, 58, 59, 101–107]. These devices include FETs, diodes and optical switches [60, 68, 108–116]. This proposed work is one approach to make a bridge between the biomolecules with III–V semiconductor technology. The heterostructure of biomolecules and III–V nanocrystalline materials can also be designed using the first-principle theoretical approach. Furthermore, electrical and optical properties of nitrogen and gold co-doped graphene are investigated using first-principle formalisms. First-principle formalism is used to find out the change of quantum–mechanical characteristics and investigation of various electronic or optical properties of the organic as well as inorganic molecules. Investigation can also be done for vacancy-defected graphene and Mn-doped graphene toward the H_2_S absorption. Ferromagnetism investigation using the first-principle approach for transition-metal doped AlN monolayer is also an emerging trend. Doping effect is investigated for monolayer MoS_2_ using DFT for visible light is an important topic of discussion. A study of change of electronic properties was demonstrated for Eu-doped phosphorene based on the first-principle approach. Electromechanical quantum transport features are available for these devices [117–121].

**Fig. 3:**
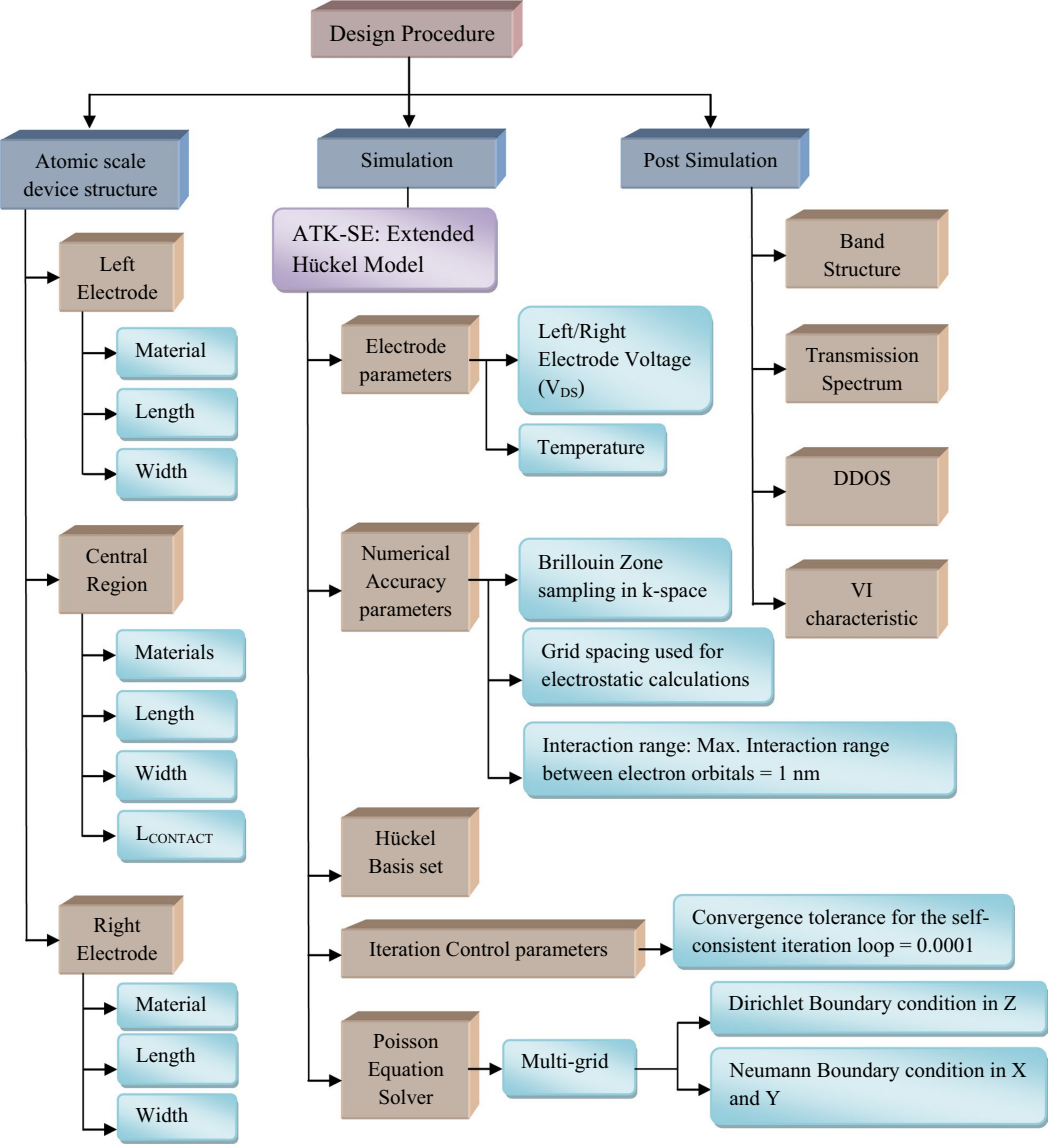
Working flowchart diagram of Quantumwise ATK-VNL [76]

In the year of 1987, Destefanis proposed the electrical doping of HgCdTe using ion implantation and heat treatment method. To increase a large number of pixels into the focal plane array devices, infrared photovoltaic detectors were required. The use of ion implanting HgCdTe was increasing this interest of manufacturers. In this type of manufacturing of photovoltaic infrared detectors, the electrical doping process was introduced. It was revealed that the effect of electrical doping into HgCdTe appeared significantly as the intrinsic properties of diodes were directly related to it [122]. Electrical was also proposed for enhancement of plasmonic absorption on Au-PbS core–shell nanocrystals. This method of doping was implemented using the intra-particle charge transfer method. In this experiment, colloidal nanocrystals were used to be the basic building blocks for solar cells, photo-detectors, etc. In this approach, researchers investigated the electronic properties of colloidal nanocrystalline materials and they also proposed a novel approach to electrical doping to these nanocrystalline solids using intra-particle charge transfer method [123]. The process flow for this simulation work is shown in Fig. 3.

## Simulation Methods of Electrical Doping

The analytical design of these molecular devices requires constant innovation and improvement in the field of material science. Density functional theory (DFT) and non-equilibrium Green’s function (NEGF) are the two key formalisms behind the analytics for the modeling of these nanoscale devices. The first-principle approach combines these two formalisms to describe theoretically these types of nanodimension devices. Extended Hückel theory (EHT) is another key factor to accelerate the design procedure of these atomistic devices [126, 127]. These theoretical modeling procedures help to prevent various problems regarding the nanoscale design like hazards during doping of foreign particles, generation of THz operating frequency, etc. Another aim of this nanoscale design procedure is to operate the device by keeping the electronic temperature at 300 K, i.e., room temperature. III–V semiconductors are optically sound semiconductor material that can be used for the design of various electronic devices. After silicon technology, III–V semiconductor technology is one of the emerging and most desirable areas to be fit in the nanoscale semiconductor technology. Biomolecules (like adenine, thymine, guanine and cytosine) have been introduced to form different nanoscale electronic devices. These biomolecules also exhibit their optical exposure whenever they are simulated at near-UV region (mid-UV-B). In this proposed work the electronic characterization has been made for the simulated nanoscale devices using the first-principle approach. This semiempirical modeling is carried out using EHT for obtaining faster simulation. We aim to design and characterize the III–V materials along with biomolecules using DFT- and NEGF-based first-principle formalisms. This semiempirical design of this bioinspired nanodevices has been carried out using the Quantumwise software simulation package.

To include electrical doping into the molecular devices, the same but opposite charge is to be provided to the two ends of the molecular interface. The electrical doping concentration is calculated using the following procedure:

Let us assume the electrodes are about 1 nm long and with 0.5 nm × 0.5 nm cross-sectional area. For simplification of calculation, we have taken those values. In the script editor, we have located the section for the electrodes calculator and assigned the charge =  + 0.01 and − 0.01. For this theoretical study, the Atomistic Tool Kit-Virtual Nano-laboratory (ATK-VNL) software package has been used. This software uses density functional theory (DFT) and non-equilibrium Green’s function (NEGF)-based first-principle approach. This value is being calculated using the following formula:

- Effective doping concentration = doping/volume [1, 70, 71]
- Assume, doping charge =  ± x V
- Assume that, volume = length (*a*) × width (*b*) × height (*c*) = *a* × *b* × *c*
- Volume = (*a* × 10^–7^) × (*b* × 10^–7^) × (*c* × 10^–7^) cm^−3^ = *abc* × 10^–21^/cm^3^
- Effective doping = $\frac{x}{{abc \times 10^{ - 21} }}$ = *abc* × 10^21^/cm^3^ [as we have consider the dimension in nm unit]

**Fig. 4:**
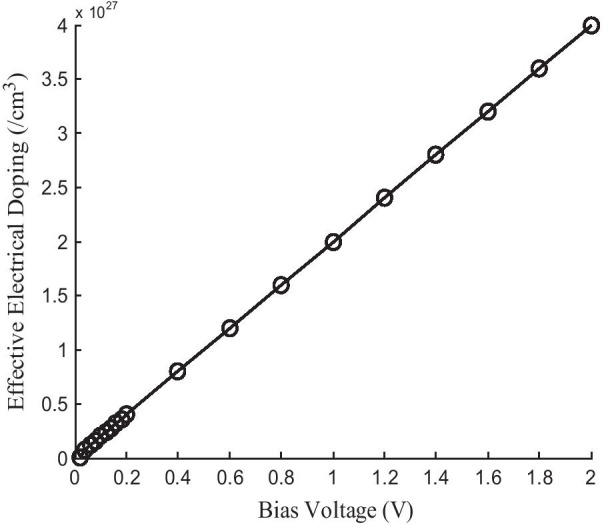
Dependence of effective electrical doping on an applied bias voltage

The volumes of the electrodes remain constant so from Fig. 4, it can be observed that the doping concentration is directly proportional to the applied bias voltage. This is another reason that we have kept constant the electrode’s size. The little change in electrodes’ size leads to a large change in electrical doping concentration. So by changing the little amount of bias voltage we can be able to generate very high electrical doping into the system using the first-principle approach.

The electrical doping in this case totally depends on two parameters mainly. They are (1) effective doping charge (charge applied at the two ends of the electrodes) and (2) volume of the nanoscale device. Therefore, the formula of calculating electrical doping is mentioned as doping/volume, so if the length, height or breadth or anyone of the parameter is changed, then the doping concentration is definitely changed. For this type of device structure, volume is a function of length, height, width [70].

Both the temperature and thickness affect the performance of these nanoscale devices. Self-heating effect along with thermal noise generated heat also makes changes in quantum-ballistic transport phenomenon of these devices at this low dimension. Therefore, temperature plays an important role in the device performance. On the other hand, as thickness is related to the volume of the device and effective doping is directly related to volume, thickness also affects device performance. If thickness is changed, then accordingly volume changes which result in changes of doping concentration. Doping concentration is related directly to device performance like channel conductivity, current–voltage characteristics, etc., for these nanoscale devices. Therefore, doping is changed due to thickness changed that will definitely change device performance [70].

## Evolution of Electrical Doping

Doping means the addition of explicit impurity atoms to the semiconductor. Doping is the intentional addition of atoms to the intrinsic semiconductor to modulate the electrical properties of intrinsic semiconductors. The electrodes sizes are inserted within the script editor, where we assigned the length of the electrode as 1 nm and cross section 0.5 nm × 0.5 nm. Thus, the nominal charge, i.e., ± 0.01, is set for the two electrodes. This script is processed through the job manager, and the calculated doping value for the electrodes is obtained. For this calculation we pursue the following steps:

- Open the New Calculator and select “ATK-SE: Extended Hückel (Device).”
- Uncheck “No SCF iteration.”
- Keep mesh cutoff to 10 Hartree.
- Under “Poisson Solver” set the “Neumann” boundary conditions along A(X) and B(Y) directions.

**Fig. 5:**
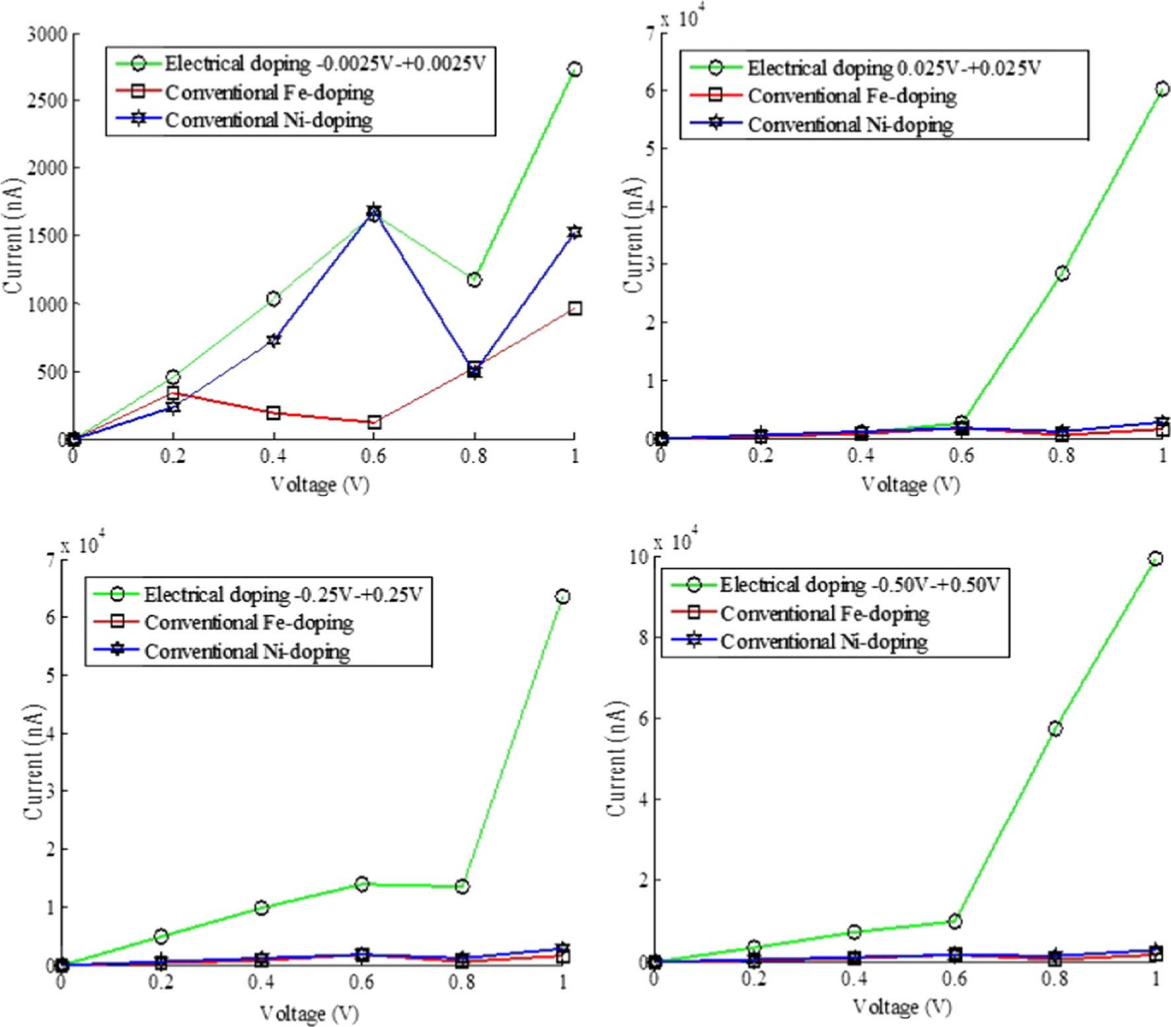
Comparative diagram at various electrical dopings along with conventional Fe- and Ni-doping

Figure 5 shows the consolidated form of the comparative study between electrical doping and conventional doping process (using Fe and Ni). This analytical experiment is observed for the thymine nanotube structure which is an example of electrical doping [70]. Fe and Ni atoms are chosen to dope the thymine nanotube, and on the other hand, the molecule is electrically doped [70]. All these results show that amount of electrical doping is much more when compared with conventional doping for little amount of applied bias. Some example works of electrical doping along with its some advantages over conventional doping are discussed in Table 2. It gives a comparative study of electrically doped devices with the existing device modeling which follows the conventional doping method. There are several types of doping, and dopants are available, for example, conventional doping (by adding impurity), electrical doping, co-doping. Generally, two types of dopants are available for conventional doping process, p-type dopants and n-type dopants. They are often called as acceptor and donor impurity atoms. These external impurities are added to the semiconducting materials to enhance their electrical properties mainly conductivity. In the case of the electrical doping process, mainly for analytical modeling using the ATK-VNL approach, we do not proceed with the addition of foreign atoms. Instead of these explicit atom doping, we focus on the change of potential difference at the two ends of the device (mainly at the ends of electrodes). The doping of a semiconductor along with another substance is known as co-doping. For example, when Co and N both are added to MoO_2_ nanowires, it will increase the electronic performance of this nanowire [128–130]. Various properties like electronic, optical and morphological characteristics of p-doped polyfuran (PF) molecular thin films were investigated by the researchers using a wide range of doping ratios using the electrical doping method. When the doping concentration is ≤ 2%, then it increased the short-circuit current of this PF-based photovoltaic device significantly [44].

**Table 2:** Comparative study of conventional doping with electrical doping

Various doped materials features	Conventional doping				Electrical doping			
	Chlorine-doped WS_2_-metal interface [131]	Co-doped ZnO structures joining the Al electrode [132]	Doped armchair graphene nanoribbons [133]	Carbon nanotubes with boron/nitrogen co-doping [52]	Electrically doped pin FET from adenine-based single-wall nanotube [55]	Electrically doped gated diode from single-wall thymine nanotube-like structure [70]	GaAs pin nanodiode [75]	Electrically doped nanobiopin FET [76]
Maximum current achieved	Data not given	− 4862 nA	6.16 × 10^−6^ A	Data not given	15.9 µA	99.3 µA	1.16 µA	35.96 nA
Composite central region length	5 × 5 WS2 super-cell (inorganic, metal interface)	Inorganic (ZnO)	Organic (graphene nanoribbon)	Organic (carbon nanotube)	Biomolecular, 3.35 nm	Biomolecular, 3.75 nm	Inorganic (GaAs), electrode is 1 nm with a cross section of 0.5 nm × 0.5 nm	Biomolecular, 6.24 nm
Operating temperature	Room temperature	Room temperature	Room temperature	Room temperature	300 K	300 K	300 K	300 K
Force tolerance	0.001 eV/Å	0.05 eV/Å	NM	0.01 eV/ºA	0.01 eV/Å	0.01 eV/Å	0.05 eV/Å	0.05 eV/Å
Applied bias (V)	–	− 1 to + 1	Low	–	0.02	0.0025–0.5	± 0.01	± 0.01
Doping concentration	2% of total sulfur atoms	2.5% of total Zn atoms	Satisfactory	B/N pairs: 5% and 10%, respectively	3.05 × 10^18^/cm^3^	5.73 × 10^18^/cm^3^	5.23 × 10^19^/cm^3^	4 × 10^19^/cm^3^

**Fig. 6:**
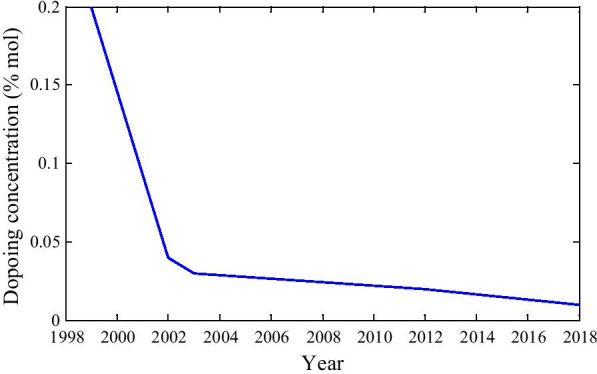
Doping concentration year-wise graph

If we take a close look at the doping concentration from Fig. 6, we can observe that before the year 2000, doping concentration was high, but after that, it becomes lower. Therefore, it can be emphasized that though the device performance has been enhanced, doping concentration is reducing very fast [124, 128, 134–136].

The optical and electrical doping process was also introduced into the silicon with holmium in the year 1999. Intermolecular hybridization state is also governed by the electrical doping process. It was established that for organic semiconductors, molecular electrical doping was found to be at odds when other methods were proved in this field, for example, the formation of polaron. Therefore, the main objective of this study is to propose a polaron-derived state with decreased ionization energy using ultraviolet photoelectrospectroscopy [134]. The electrical doping profile in ferroelectric film capacitors was investigated by the group of researchers using capacitance–voltage measurement. In this experimental study, profiling effect of electrical doping concentration in ferroelectrics was investigated using the following effects of

- A field and spatially dependent permittivity.
- Domain switching analysis of Schottky profiling [135].

**Fig. 7:**
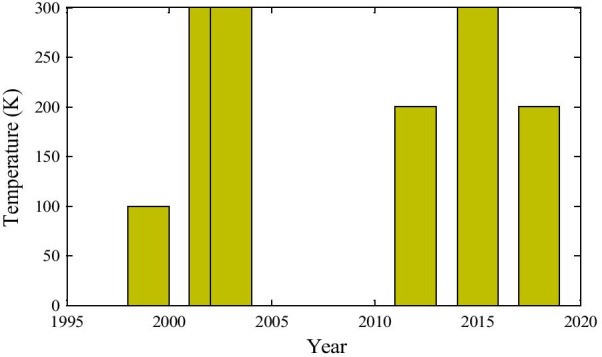
Temperature for doping

From Fig. 7, we can observe the operating temperature for this type of doping-dependent device operation. Though the graph is a little bit complex, it does not obey any specified rule. Therefore, we can conclude it like that temperature requirement is solely depending on the type of materials that are used for this operation.

A new model was proposed for the dissociation of carbon atoms at the copper/silica molecular thin layer interface using catalytically hydrogenated graphene meshes using a semipermanent electrical doping method. This process enables stable electronic doping through C–N bonds. Furthermore, the effect of trap states on the electrical doping for organic semiconductors was also investigated. The direct charge transfer process from the trap state of the host molecules to the dopant molecules raised the electrical effect for organic semiconductors. This type of doping process enhances conductivity. Therefore, trap density and energy are also analyzed using impedance spectroscopy [136].

**Fig. 8:**
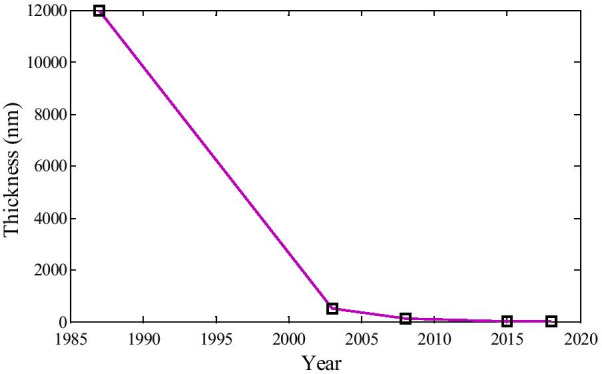
The thickness of the wafer

It is observed clearly from Fig. 8 that the thickness of the wafer layers is reducing year wise. The more the time increasing, the layer thickness reduces, and the performance of the device increases.

Electrically doped and undoped poly(9,9-dioctylfluorenyl-2,7-diyl) (PFO) along with tetrafluorotetracyanoquinodimethane films were composed using photoelectron spectroscopy method and also investigated their current–voltage characteristics. Thus, it can be observed that the depletion region was created for the PFO interface. Therefore, the current was increased subsequently [137, 138]. For high-temperature gas sensors, this method of doping plays an important role. The conductivity and gas sensitivity of Ga_2_O_3_ thin films was investigated. It was observed that this doping concentration influenced the surface sensitivity [138].

**Fig. 9:**
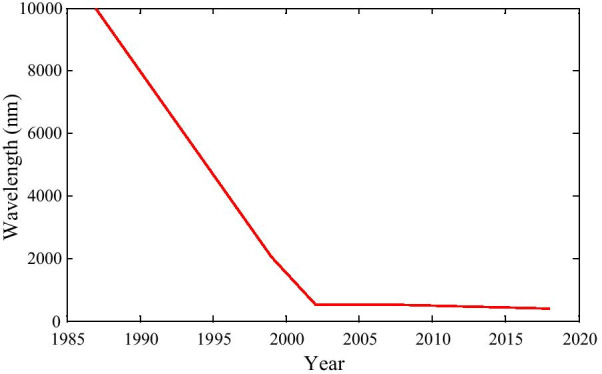
The wavelength of the devices reduces

From Fig. 9, it is observed that the cutoff wavelength of the devices reduces sharply within a few decades. Hence, device performance enhanced significantly. Table 3 gives a close look at different characteristics of the devices which follow either electrical doping or conventional doping procedure.

**Table 3:** Several properties of the devices with conventional doping and electrical doping approaches at a glance

Article with Ref. no. features	Electrical doping approach			Conventional doping approach		
	Yu et al. [44]	Lee et al. [123]	Tietze et al. [124]	Ling et al. [48]	An et al. [49]	Liu et al. [97]
Device	Polyfuran-based photo-voltaic cells	Au-PbS core–shell nanocrystals	OLEDs	Triangular grapheme with B/N doped	Graphene nanoribbon	Copper-modified DNA
Conductivity	High	1 S/cm	> 10^–2^ S/cm	–	–	Enhanced
Driving voltage	Low open-circuit voltage 0.2–0.4 V	− 40 to + 40 V	− 2.0 < V < 0.3	− 2.2 to 2.2	–	− 0.6 to + 0.6 V
Doping	Dopant concentration ≤ 2%	High doping density	0.1 mol%	B/N doped	B/N doped	Cu doped
Procedure	Atomic force microscopy	Intra-particle charge transfer (plasmonic enhancement)	Ground state integer charge transfer	NEGF + DFT	DFT + NEGF	DFT + NEGF
Constrain	Enhanced work function at high dopant concentration cannot be explained by integer charge transfer	The amount of charge transferred between Au and PbS depends on the core size and shell thickness which still has to be determined	Electrical doping is not sufficient to fill the deep traps	Intra-molecular weak interaction effect on rectifying property	Theoretical approach	Theoretical approach

In this survey, we have reviewed the works which were already established using the electrical doping process. In our works, we used the electrical doping process using the Quantumwise software simulation package in the ATK-VNL atmosphere. The version of this software is 13.8.0. This software simulation is based on first-principle formalisms which is again strongly supported by DFT and NEGF formalisms. Quantumwise is a compact set of atomic-scale modeling tools. These tools were developed in the year of 2003 by some software professionals along with academicians. These ATK-VNL simulations engines help us to calculate the electronic structure as well as to formulate intercorrelations of atomic orbitals. This platform helps us to introduce electrical doping into the molecular level.

## Conclusion

This report illustrates briefly a comparison between conventional doping and electrical doping process. Though the electrical doping process is not so newer process, the implementation of this process with the help of DFT- and NEGF-based first-principle approach gives a new twist to this phenomenon. Therefore, electrical doping is to be implemented in many molecular modeling approaches to bring a new era in nanoelectronics. This study takes a close look at the electrical doping phenomenon such as why it is important, how it works for the molecular modeling approach, calculation of electrical doping concentration, etc. Hence, we provide a comparative study between electrical doping and conventional doping process for acepromazine molecule. To conclude it is emphasized that in future this is one of the approaches which will prove itself in the field of nanodevice modeling.

## Data Availability

All the data and material are available in the manuscript.

## Abbreviations

DFT: Density functional theory
NEGF: Non-equilibrium Greens’ function
OPVC: Organic photovoltaic cell
ATK-VNL: Atomistix Tool Kit-Virtual Nano-Laboratory
HOMO–LUMO: Highest occupied molecular orbital–lowest unoccupied molecular orbital
CNT: Carbon nanotube
I–V: Current–voltage
QCA: Quantum cellular automata
YN_2_: Yttrium nitrite
ATK-SE: Atomistix Tool Kit-Semi-empirical
